# Diagnostic accuracy of stroke volume variation measured with uncalibrated arterial waveform analysis for the prediction of fluid responsiveness in patients with impaired left ventricular function: a prospective, observational study

**DOI:** 10.1007/s10877-015-9743-2

**Published:** 2015-07-31

**Authors:** L. J. Montenij, J. P. C. Sonneveld, A. P. Nierich, W. F. Buhre, E. E. C. de Waal

**Affiliations:** 1 Department of Anaesthesiology, Intensive Care and Emergency Medicine, University Medical Centre Utrecht, Heidelberglaan 100, 3584 CX Utrecht, The Netherlands; 2 Department of Anaesthesia and Intensive Care, Isala Clinics, Dokter van Heesweg 2, 8025 AB Zwolle, The Netherlands; 3 Department of Anaesthesia and Pain Therapy, Maastricht University Medical Centre, Debeyelaan 25, 6229 HX Maastricht, The Netherlands

**Keywords:** Cardiac preload, Dynamic preload, Intraoperative monitoring, Pulse wave analysis, Heart failure

## Abstract

Uncalibrated arterial waveform analysis enables dynamic preload assessment in a minimally invasive fashion. Evidence about the validity of the technique in patients with impaired left ventricular function is scarce, while adequate cardiac preload assessment would be of great value in these patients. The aim of this study was to investigate the diagnostic accuracy of stroke volume variation (SVV) measured with the FloTrac/Vigileo™ system in patients with impaired left ventricular function. In this prospective, observational study, 22 patients with a left ventricular ejection fraction of 40 % or less undergoing elective coronary artery bypass grafting were included. Patients were considered fluid responsive if cardiac output increased with 15 % or more after volume loading (7 ml kg^−1^ ideal body weight). The following variables were calculated: area under the receiver operating characteristics (ROC) curve, ideal cut-off value for SVV, sensitivity, specificity, positive and negative predictive values, and overall accuracy. In addition, SVV cut-off points to obtain 90 % true positive and 90 % true negative predictions were determined. ROC analysis revealed an area under the curve of 0.70 [0.47; 0.92]. The ideal SVV cut-off value was 10 %, with a corresponding sensitivity and specificity of 56 and 69 % respectively. Overall accuracy was 64 %, positive and negative predictive values were 69 and 56 % respectively. SVV values to obtain more than 90 % true positive and negative predictions were 16 and 6 % respectively. The ability of uncalibrated arterial waveform analysis SVV to predict fluid responsiveness in patients with impaired LVF was low.

## Introduction

Volume loading remains one of the cornerstones in the treatment of critically ill patients in the intensive care unit and operating room. Although fluid therapy may prevent tissue ischaemia, excessive use of fluids is associated with increased mortality and morbidity [1–3]. In patients with impaired left ventricular function (LVF), cardiac decompensation may easily occur. In contrast, withholding fluids may further decrease cardiac output (CO), leading to a low CO state and impaired tissue perfusion. This emphasizes the need for adequate assessment of cardiac preload in these patients.

The static filling pressures central venous pressure (CVP) and pulmonary capillary wedge pressure (PCWP) are still frequently used for this purpose, although these parameters fail to predict fluid responsiveness reliably [4]. Moreover, pulmonary artery catheterization is rather invasive. Dynamic preload assessment with uncalibrated arterial waveform analysis provides a less invasive alternative [5, 6]. The dynamic preload indices stroke volume variation (SVV) and pulse pressure variation (PPV) are the result of the cyclic influence of positive pressure ventilation on stroke volume and pulse pressure in mechanically ventilated patients [7]. If a number of criteria for mechanical ventilation are met in patients without cardiac arrhythmias or sternotomy, both SVV and PPV predict fluid responsiveness reliably in a variety of patients with cut-off points varying between 10 and 15 % [5–7].

In patients with impaired LVF, the validity of dynamic preload indices is less established [7, 8]. These patients may benefit most from reliable cardiac preload assessment, preventing the unwanted effects of hypo- and hypervolaemia, and the inappropriate use of vasoactive drugs. The ideal cut-off values for SVV and PPV and response to volume loading may be different from patients with normal cardiac function, as the course of the Frank Starling curve is flattened in patients with impaired LVF. In addition, the initial increase in stroke volume (SV) as observed in the early phase of positive pressure inspiration may be more prominent, because the left ventricle may be more sensitive to the reduction in cardiac afterload if its function is impaired [5, 7]. For that reason, the results from studies in patients with normal cardiac function cannot be simply generalized to patients with impaired LVF [8]. The present technological study investigates the diagnostic accuracy of SVV measured with the FloTrac/Vigileo™ system to predict fluid responsiveness in patients with impaired LVF undergoing coronary artery bypass grafting (CABG).

## Methods

### Patients

In this prospective, observational study, patients scheduled for elective CABG with impaired LVF were included. As part of the pre-operative work-up, transthoracic echocardiography (TTE) or magnetic resonance imaging (MRI) of the heart was performed for qualitative assessment of left and right ventricular function, measurement of left ventricular ejection fraction (LVEF), and quantitative assessment of valvular function. Patients with a LVEF ≤ 40 % were eligible [9]. Exclusion criteria were significant valvular heart disease (tricuspid, pulmonary, mitral and/or aortic valve stenosis and/or insufficiency grade ≥ 2), right ventricular dysfunction, intracardiac shunts, cardiac arrhythmias, age below 18, and patients undergoing emergency surgery. Intra-operatively, transesophageal echocardiography (TEE) was performed, to verify the presence of impaired LVF and the absence of valvular heart disease and right ventricular dysfunction.

### Anaesthesia protocol

Patients were premedicated with oral midazolam 7.5 mg 1 h before surgery. Before induction of anaesthesia, a radial artery catheter was inserted and connected to the FloTrac™ sensor and Vigileo™ monitor (Edwards Lifesciences, Irvine, CA, software version 3.02) for continuous measurement of SVV [10, 11]. The transducer was adjusted to the level of the right atrium. As previously described, positive pressure ventilation induces a cyclic change in SV, and SVV is calculated from the minimal and maximal SV using the formula [7, 11]:

$$SVV \left( \% \right) = 100 \times \frac{{SV_{max} - SV_{min} }}{{\left( {SV_{max} + SV_{min} } \right) / 2 }}$$

General anaesthesia was induced with sufentanil (0.125 μg kg^−1^) and midazolam (0.075 mg kg^−1^). Orotracheal intubation was facilitated using rocuronium bromide (1.0 mg kg^−1^). Anaesthesia was maintained using 0.5–1.0 minimum alveolar concentration of Sevoflurane. Mechanical ventilation was performed with a maximum breathing frequency of 15 min^−1^, and a tidal volume of 8 ml kg^−1^ ideal body weight. Ideal body weight (IBW) was calculated with the formula IBW = 22 * (body length [m])^2^. The inspired oxygen fraction was 0.4–0.5, with a positive end-expiratory pressure of 5–10 cm H_2_O. Pulmonary artery catheterization was performed via the internal jugular vein, guided by typical pressure waveform changes (Swan-Ganz CCOmbo™ catheter type 744HF75, Edwards Lifesciences, Irvine, CA, USA). A TEE probe was introduced.

### Data collection

Between induction of anaesthesia and incision, volume loading with 7 ml kg^−1^ (IBW) crystalloid fluid was performed in 15 min. Intermittent thermodilution cardiac output (TDCO) was measured before and after volume loading, together with heart rate (HR), mean arterial blood pressure (MAP), CVP, PCWP, and SVV. In case of administration or use of short-acting vasoactive drugs, measurements were postponed until haemodynamic stability was restored. TDCO represents the average of 5 bolus injections of 10 ml saline at room temperature, randomly spread over the respiratory cycle, and performed by the same, experienced observer [12]. At the moment of injection for a single TDCO measurement, SVV was recorded. SVV represents the average of five readings at the same time as the injections for TDCO measurement.

### Analysis

Statistical analysis was performed using SPSS version 21.0 for Windows XP (SPSS Inc, Chicago, IL, USA). We hypothesized that SVV reliably predicts fluid responsiveness in patients with impaired LVF, resulting in an area under the curve (AUC) as determined by receiver operating characteristics (ROC) analysis of ≥0.80. In a previous study by our group investigating SVV in patients with a normal LVF undergoing CABG, a sample size of 22 patients was sufficient to obtain an AUC > 0.80, with the lower 95 % CI bound > 0.70 [13]. Data are expressed as mean ± standard deviation (SD) unless otherwise stated. Possible differences between haemodynamic variables in fluid responders and non-responders were investigated using the Mann–Whitney U test. The Wilcoxon signed-rank test was used to detect possible differences before and after volume loading. Precision of TDCO is defined as twice the SD of the repeated measurements divided by √5 times TDCO (2 · SD/√5 · TDCO) [14]. Precision of TDCO was expected to be approximately 10 %, which is sufficiently precise to detect a change in CO (∆CO) of 14.1 % (√2 * 10 %) [14]. To distinguish fluid responders from non-responders, a ∆CO of ≥15 % after volume loading was used. ROC analysis was performed to quantify the prediction of fluid responsiveness for SVV. Pearson correlation analysis was used to investigate the relation between SVV before volume loading and ∆CO. For the range of SVV values before volume loading, the sensitivity (Se) and specificity (Sp) were calculated. The ideal cut-off point for SVV was defined as the SVV value corresponding to the maximum sum of Se and Sp, in which both Se and Sp were ≥50 %. For the ideal SVV cut-off, the positive predictive value (PV+), negative predictive value (PV−), and overall accuracy were calculated. Overall accuracy was defined as the proportion of correct classifications of fluid responders and non-responders, which is the sum of true positives and true negatives divided by the number of patients [15]. In addition, the SVV cut-off values to obtain a PV+ and PV− of ≥90 % were determined. A *p* value of 0.05 was considered statistically significant.

## Results

Between August 2012 and August 2014, 31 patients were eligible based on pre-operative TTE and MRI. Three patients were pre-operatively excluded: 2 for logistical reasons, and 1 patient turned out to need emergency surgery. Intra-operatively, 6 additional patients were excluded: 3 due to improvement in LVF (LVEF > 40 %), 2 because of mitral valve insufficiency grade ≥2, and 1 patient with newly developed atrial fibrillation. As a result, 22 patients were available for data analysis.

Patient characteristics, including history and medication, and pre-operative LVEF data are presented in Table 1. All patients showed sinus rhythm, except for 1 patient who had a pacemaker (atrioventricular sequential pacing mode). The overall precision for measurement of CO was 7.3 %. Relevant haemodynamic variables before and after volume loading are depicted in Table 2. PCWP measurements were not performed in three cases at the discretion of the attending anaesthesiologist. Volume loading induced significant changes in all haemodynamic variables. In Table 3, the haemodynamic variables before volume loading are presented, for responders (9 patients) and non-responders (13 patients) separately. SVV was the only haemodynamic variable that was significantly different between responders and non-responders.

**Table 1:** Patient characteristics, comorbidity and medication

	Mean (SD)	Range
Age (years)	65 (13)	42–85
Height (m)	1.75 (0.08)	1.60–1.95
Weight (kg)	85.6 (11.1)	67.0–113
BMI (kg m^−2^)	27.9 (3.4)	23.8–36.5
LVEF (%)	29.8 (7.1)	17–40

	Number of patients
Patients with	
35 % > LVEF ≤ 40 %	4
30 % > LVEF ≤ 35 %	5
25 % > LVEF ≤ 30 %	5
20 % > LVEF ≤ 25 %	6
15 % > LVEF ≤ 20 %	2
Gender	
Male/female	18/4
Comorbidity	
Diabetes mellitus	10
Hypertension	20
COPD	4
Dyslipidemia	15
Medication	
Beta blocker	20
Calcium blocker	2
ACE or AR inhibitor	17
Diuretics	13
Nitrates	5

*SD* standard deviation, *BMI* Body Mass Index, *LVEF* left ventricular ejection fraction, *COPD* chronic obstructive pulmonary disease, *ACE* angiotensin converting enzyme, *AR* angiotensin receptor

**Table 2:** Mean and SD of haemodynamic variables heart rate (HR), mean arterial pressure (MAP), central venous pressure (CVP), pulmonary capillary wedge pressure (PCWP), thermodilution cardiac output (TDCO), thermodilution stroke volume (TDSV), and stroke volume variation (SVV), for pooled data, and precision of TDCO before and after volume loading (VL)

Haemodynamic variable	Before VL	After VL
HR (beats min^−1^)	62 (13)	55 (13)*
MAP (mm Hg)	70 (13)	75 (13)*
CVP (mm Hg)	10 (2.9)	12 (3.2)*
PCWP (mm Hg)	13 (2.9)	16 (3.2)*
TDCO (L min^−1^)	3.4 (0.8)	3.8 (1.0)*
TDSV (mL beat^−1^)	56 (15)	63 (13)*
SVV (%)	11 (4.7)	8.3 (3.6)*

* Statistically significant difference (*p* < 0.05)

**Table 3:** Mean and SD of haemodynamic variables before volume loading, for responders and non-responders

Haemodynamic variable	Responders (n = 9)	Non-responders (n = 13)
HR (beats min^−1^)	66 (13)	58 (12)
MAP (mm Hg)	70 (18)	69 (7.5)
CVP (mm Hg)	9.8 (3.3)	10 (2.7)
PCWP (mm Hg)	12 (3.3)	14 (2.5)
TDCO (L min^−1^)	3.3 (1.0)	3.4 (0.6)
TDSV (mL beat^−1^)	49 (13)	61 (15)
SVV (%)	13 (5.3)	9.2 (3.7)*

* Statistically significant difference (*p* < 0.05)

**Fig. 1:**
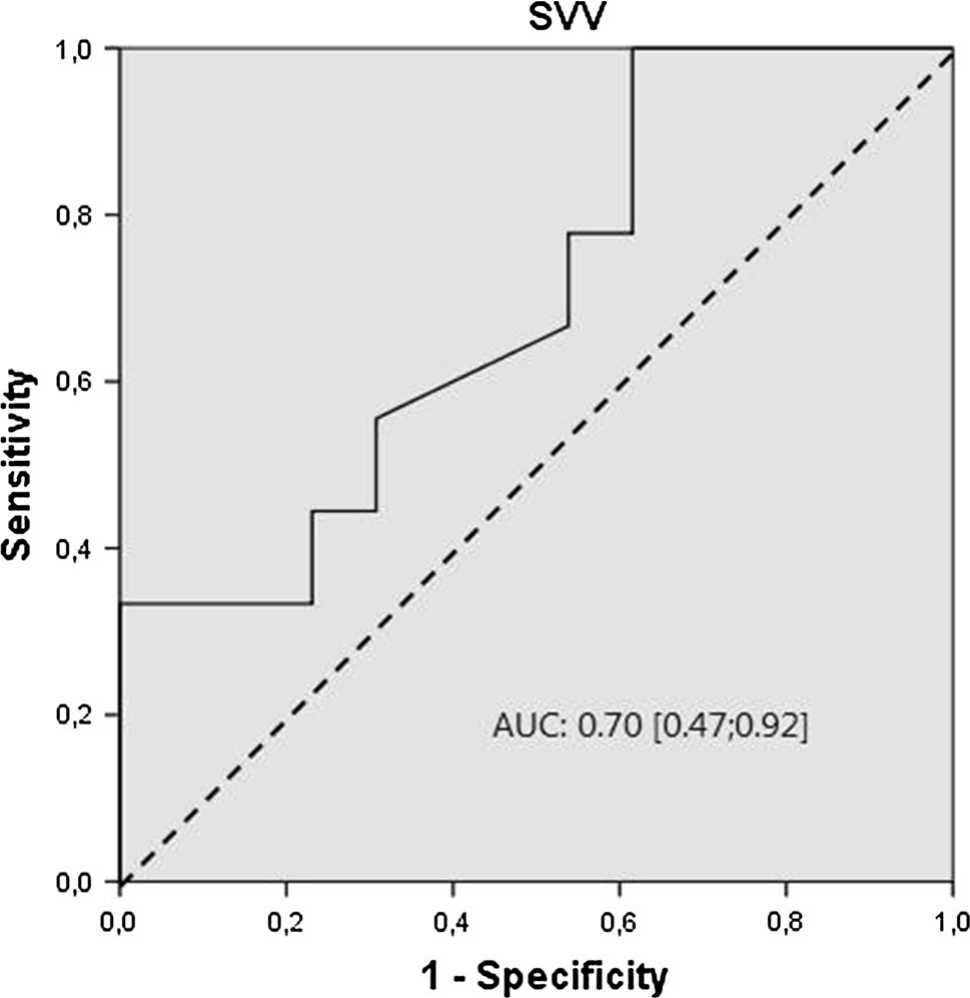
ROC curve for SVV. The *dotted line* represents the “line of no-discrimination”, indicating random guessing. The AUC for SVV and corresponding 95 % CI are presented

**Fig. 2:**
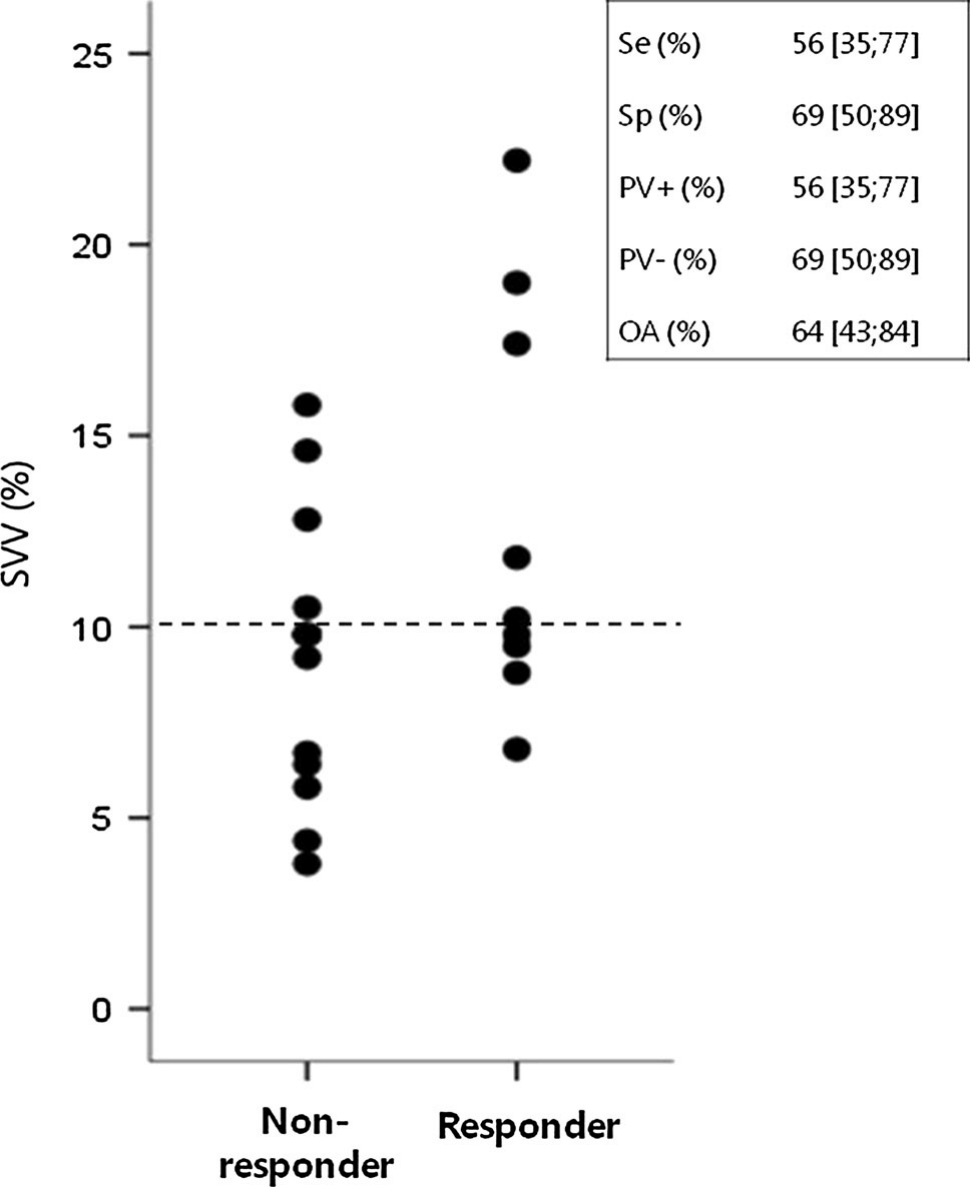
SVV in responders and non-responders. Ideally, a *horizontal line* can be drawn which separates fluid responders from non-responders. The *dotted line* depicts the 10 % SVV cut-off, the corresponding Se, SP, PV+, PV− and overall accuracy (OA) are presented, including their 95 % CIs

In Fig. 1, the results from ROC analysis are presented. The AUC for SVV was 0.70, but did not differ significantly from 0.5 (95 % CI [0.47; 0.92]). Figure 2 displays the SVV values for fluid responders and non-responders. The highest sum of Se and Sp corresponded to a SVV cutt-off of 10 % (dotted line). The Se, SP, PV+, PV− and overall accuracy for this 10 % cut-off are shown in the box in Fig. 1. The overall accuracy, PV+, and PV− were lower than 90 %, including the upper level of the 95 % CI. The SVV cut-off value to obtain a PV+ of ≥90 % was 16 %, whereas a SVV cut-off value of 6 % was needed to obtain a PV− of ≥90 %. The Pearson correlation coefficient for SVV and ∆CO was 0.32 (*p* = 0.16).

## Discussion

In the present study, we investigated the diagnostic accuracy of SVV measured with the FloTrac/Vigileo™ system to predict fluid responsiveness in patients with a LVEF of ≤40 % undergoing elective CABG. The predictive value of SVV found in this study was lower in comparison with the results from a systematic review and meta-analysis and in individual trials [6, 8].

The validity of dynamic preload variables has been thoroughly studied in the past decades, but evidence in patients with impaired cardiac function is limited [6–8, 16, 17]. The aim of the present study was therefore to add new information, as in these patients, fluid balance is delicate and the chance of cardiac failure due to inappropriate volume treatment is increased. In analogy with a previous study of Reuter and co-workers, the predictive value of SVV was decreased compared to a patient population with normal cardiac function as reflected by an AUC of only 0.7 in the ROC analysis. The results may be explained by a flattened course of the Frank Starling curve in patients with impaired LVF. In this situation, SV is most likely less dependent on the cyclic changes in intrathoracic pressure as changes in preload may only lead to small changes in SV. Moreover, in some patients it can be expected that volume load results in hypervolaemia and thus decreases in SV can be expected. These effects hinder discrimination of fluid responders from non-responders, partly explaining the decreased diagnostic accuracy.

A second relevant finding of this study is the fact that wide confidence intervals were present. The 95 % CI of the AUC as determined by ROC analysis was [0.47; 0.92], resulting in a total 95 % CI width of 0.45. In a previous study by our group involving 22 patients with normal cardiac function, the 95 % CI bounds were [0.78; 1.0], with a total 95 % CI width of 0.22 [13]. This is in line with the study by Reuter [8]. In their study, the 95 % CI of the AUC was [0.59; 0.96], with a total 95 % width of 0.37 for patients with a LVEF < 35 %, versus a 95 % CI of [0.77; 0.99] and a total width of 0.22 for patients with a LVEF > 50 %. Most likely, patients with impaired LVF show a much more variable response to a given volume challenge compared to patients with normal cardiac function. This finding can, at least, partly explain the wide 95 % CIs and thus the lack of significance when using SVV as a predictor of volume responsiveness.

Apart from this physiological explanation, lack of diagnostic accuracy could be the result of inconsistencies of the SVV measurement technique. Although uncalibrated arterial waveform analysis SVV is a valuable technique for predicting fluid responsiveness in a variety of patients, the validity of the technique may be impeded in patients with impaired LVF. Calibrated arterial waveform analysis techniques provide more stable measurement of SV [7, 16]. However, SVV is the result of the cyclic changes in SV, not its absolute value. Question remains therefore whether the use of calibrated techniques improve the accuracy of SVV in patients with impaired LVF. Reuter and co-workers applied a calibrated technique, and the results are comparable to the results of the current trial [8]. This indicates that the use of an uncalibrated technique does not fully explain the results from the present study.

The present study has a number of strengths. All patients met the prerequisites for dynamic preload assessment with respect to mechanical ventilation and closed chest conditions [5, 13, 18, 19]. Volume loading induced significant changes in haemodynamic variables, suggesting that a 7 ml kg^−1^ (IBW) fluid challenge was sufficient. Precision of CO measurement was 7.3 % on average, which indicates that fluid responders were reliably discriminated from non-responders using a 15 % CO increase. Overall accuracy and positive and negative predictive values were included in the data analysis. These prediction variables directly inform clinicians about the risk of incorrect use of fluid support. Moreover, the SVV cut-off values corresponding to a PV+ and PV− ≥90 % were determined. The choice of a single SVV cut-off value is doubtful as it frequently turns out to be both insufficiently sensitive and insufficiently specific [15, 20]. In our patients, SVV needed to be ≥16 % to limit the risk of unnecessary volume loading to <10 %, and SVV ≤ 6 % was the threshold to withhold unwanted withholding fluids to <10 %. This implies that a broad range of SVV values could not be used for clinical decision making.

A limitation of the study is the relatively small number of patients. In order to obtain a homogeneous selection, patients with cardiac pathology other than a LVEF ≤ 40 % were excluded and measurements were restricted to the period before incision. A major drawback of this approach is the limited availability of eligible patients. Despite the limited number of patients, the overall conclusion of this study seems valid. The upper 95 % CI levels for PV+ (77 %) and PV− (89 %) indicate a minimal false positive rate of 23 %, and a false negative rate of 11 %. We did not compare SVV with other preload variables like PPV, which is another limitation. Studies comparing PPV measurements with SVV in patients with impaired LVF are most likely helpful to discriminate between pathophysiological effects or methodological topics as an explanation for our results. In addition, the use of calibrated SVV techniques, a multi-centre approach, extension of the inclusion criteria, or multiple measurements per patient might be considered for future research.

In conclusion, the results from this study showed that the diagnostic accuracy of uncalibrated SVV to predict fluid responsiveness in patients with impaired LVF was low. Therefore, the uncritical use of SVV has a high risk of making incorrect decisions in this vulnerable patient group.

## References

[1] Bundgaard-Nielsen M, Secher NH, Khelet H (2009). ’Liberal’ vs. ‘restricted’ perioperative fluid therapy—a critical assessment of the evidence. Acta Anaesthesiol Scand.

[2] Wiedemann HP, Wheeler AP, Bernard GR, Thompson BT, Hayden D, De Boisblanc B, Connors AF, Hite RD, Harabin AL (2006). Comparison of two fluid-management strategies in acute lung injury. N Engl J Med.

[3] Silva JM, de Oliveira AM, Noqueira FM, Vianna PM, Pereira Filho MC, Dias LF, Maia VP, Neucamp CD, Amendola CP, Carmona MJ, Malbouisson LM (2013). The effect of excess fluid balance on the mortality rate of surgical patients: a multicenter prospective study. Crit Care.

[4] Marik PE, Baram M, Vahid B (2008). Does central venous pressure predict fluid responsiveness? A systematic review of the literature and the tale of seven mares. Chest.

[5] Michard F, Teboul JL (2000). Using heart-lung interactions to assess fluid responsiveness during mechanical ventilation. Crit Care.

[6] Marik PE, Cavalazzi R, Vasu T, Hirani A (2009). Dynamic changes in arterial waveform derived variables and fluid responsiveness in mechanically ventilated patients: a systematic review of the literature. Crit Care.

[7] Montenij LJ, De Waal EE, Buhre WF (2011). Arterial waveform analysis in anesthesia and critical care. Curr Opin Anaesthesiol.

[8] Reuter DA, Kirchner A, Felbinger TW, Weis FC, Kilger E, Lamm P, Goetz E (2003). Usefulness of left ventricular stroke volume variation to assess fluid responsiveness in patients with reduced cardiac function. Crit Care Med.

[9] Yancy CW, Jessup M, Bozkurt B (2013). 2013 ACCF/AHA guideline for the management of heart failure: a report of the American College of Cardiology Foundation/American Heart Association Task Force on Practice Guidelines. Circulation.

[10] Manecke GR (2005). Edwards FloTrac sensor and Vigileo monitor: easy, accurate, reliable cardiac output assessment using the arterial pulse wave. Expert Rev Med Devices.

[11] Hashim B, Lerner AB (2010). The FloTrac™ System—measurement of stroke volume and the assessment of dynamic fluid loading. Int Anesthesiol Clin.

[12] Jansen JR, Schreuder JJ, Settels JJ, Kloek JJ, Versprille A (1990). An adequate strategy for the thermodilution technique in patients during mechanical ventilation. Intensive Care Med.

[13] De Waal EE, Rex S, Kruitwagen CL, Kalkman CJ, Buhre WF (2009). Dynamic preload indicators fail to predict fluid responsiveness in open chest conditions. Crit Care Med.

[14] Cecconi M, Rhodes A, Poloniecki J, Della Rocca G, Grounds RM (2009). Bench-to-bedside review: the importance of the precision of the reference technique in method comparison studies—with specific reference to the measurement of cardiac output. Crit Care.

[15] Linnet K, Bossuyt PM, Moons KG, Reitsma JB (2012). Quantifying the accuracy of a diagnostic test or marker. Clin Chem.

[16] Alhashemi JA, Cecconi M, Hofer CK (2011). Cardiac output monitoring: an integrated perspective. Crit Care.

[17] Cannesson M, Pestel G, Ricks C, Hoeft A, Perel A (2011). Hemodynamic monitoring and management in patients undergoing high risk surgery: a survey among North American and European anesthesiologists. Crit Care.

[18] Heenen S, De Backer D, Vincent JL (2006). How can the response to volume expansion in patients with spontaneous respiratory movements be predicted?. Crit Care.

[19] Reuter DA, Bayerlein J, Goepfert MS, Weis FC, Kilger E (2003). Influence of tidal volume on left ventricular stroke volume variation measured by pulse contour analysis in mechanically ventilated patients. Intensive Care Med.

[20] Feinstein AR (1990). The inadequacy of binary models for the clinical reality of three-zone diagnostic decisions. J Clin Epidemiol.

